# Phospho-heavy-labeled-spiketide FAIMS stepped-CV DDA (pHASED) provides real-time phosphoproteomics data to aid in cancer drug selection

**DOI:** 10.1186/s12014-022-09385-7

**Published:** 2022-12-19

**Authors:** Dilana E. Staudt, Heather C. Murray, David A. Skerrett-Byrne, Nathan D. Smith, M. Fairuz B. Jamaluddin, Richard G. S. Kahl, Ryan J. Duchatel, Zacary P. Germon, Tabitha McLachlan, Evangeline R. Jackson, Izac J. Findlay, Padraic S. Kearney, Abdul Mannan, Holly P. McEwen, Alicia M. Douglas, Brett Nixon, Nicole M. Verrills, Matthew D. Dun

**Affiliations:** 1 School of Biomedical Sciences and Pharmacy, College of Health, Medicine and Wellbeing, University of Newcastle, Callaghan, NSW 2308 Australia; 2 Precision Medicine Research Program, Hunter Medical Research Institute, New Lambton Heights, NSW 2305 Australia; 3 School of Environmental and Life Sciences, College of Engineering, Science and Environment, University of Newcastle, Callaghan, NSW 2308 Australia; 4 Infertility and Reproduction Research Program, Hunter Medical Research Institute, New Lambton Heights, NSW 2305 Australia; 5 Analytical and Biomolecular Research Facility (ABRF), Research Services, University of Newcastle, NSW, Callaghan, 2308 Australia

**Keywords:** Phosphoproteomics, Drug targets, Oncogenic signaling, Acute myeloid leukemia, Cancer, pHASED, ATM, Clinical phosphoproteomics, Resistance, Combination therapy

## Abstract

Global high-throughput phosphoproteomic profiling is increasingly being applied to cancer specimens to identify the oncogenic signaling cascades responsible for promoting disease initiation and disease progression; pathways that are often invisible to genomics analysis. Hence, phosphoproteomic profiling has enormous potential to inform and improve individualized anti-cancer treatment strategies. However, to achieve the adequate phosphoproteomic depth and coverage necessary to identify the activated, and hence, targetable kinases responsible for driving oncogenic signaling pathways, affinity phosphopeptide enrichment techniques are required and often coupled with offline high-pressure liquid chromatographic (HPLC) separation prior to nanoflow liquid chromatography–tandem mass spectrometry (nLC-MS/MS). These complex and time-consuming procedures, limit the utility of phosphoproteomics for the analysis of individual cancer patient specimens in real-time, and restrict phosphoproteomics to specialized laboratories often outside of the clinical setting. To address these limitations, here we have optimized a new protocol, phospho-heavy-labeled-spiketide FAIMS Stepped-CV DDA (pHASED), that employs online phosphoproteome deconvolution using high-field asymmetric waveform ion mobility spectrometry (FAIMS) and internal phosphopeptide standards to provide accurate label-free quantitation (LFQ) data in real-time. Compared with traditional single-shot LFQ phosphoproteomics workflows, pHASED provided increased phosphoproteomic depth and coverage (phosphopeptides = 4617 pHASED, 2789 LFQ), whilst eliminating the variability associated with offline prefractionation. pHASED was optimized using tyrosine kinase inhibitor (sorafenib) resistant isogenic FLT3-mutant acute myeloid leukemia (AML) cell line models. Bioinformatic analysis identified differential activation of the serine/threonine protein kinase ataxia-telangiectasia mutated (ATM) pathway, responsible for sensing and repairing DNA damage in sorafenib-resistant AML cell line models, thereby uncovering a potential therapeutic opportunity. Herein, we have optimized a rapid, reproducible, and flexible protocol for the characterization of complex cancer phosphoproteomes in real-time, a step towards the implementation of phosphoproteomics in the clinic to aid in the selection of anti-cancer therapies for patients.

**Supplementary Information:**

The online version contains supplementary material available at 10.1186/s12014-022-09385-7.

## Introduction

Mass spectrometry based approaches for global high-throughput quantitation of cellular phosphoproteomes have been increasingly applied to cancer specimens as they provide powerful tools for the identification of signaling pathways including kinases, phosphatases, tumor suppressor and cell cycle regulators that drive disease initiation and progression [1–4]. Deregulation of kinase activity plays a critical role in cancer development and relapse [5–8], highlighting kinases as important therapeutic targets in the clinic [9, 10]. This is particularly the case for FLT3 kinase-driven acute myeloid leukemia (AML) patients. The FMS-like tyrosine kinase 3 (FLT3) receptor tyrosine kinase is recurrently mutated in AML and is the target for FLT3 inhibitors. The most common mutations are internal tandem duplications (ITD) and kinase domain mutations (e.g., D835). Resistance to FLT3 inhibitors is often associated with the emergence of dual FLT3-ITD/D835 mutations, however the pathways upregulated during the development of resistance are yet to be fully characterized [3, 6, 7]. Therefore, phosphoproteomic profiling of patients’ specimens in real-time to identify the activated kinases responsible for driving downstream oncogenic signaling cascades, provides us with the opportunity to repurpose clinically relevant therapeutics [11–15], and thus aid in the development of personalized treatment strategies that may improve overall survival.

Several methods have been developed for the quantitative characterization of phosphoproteins in complex biological samples using shotgun proteomics [16–19]. Stable isotope-labeling strategies such as tandem mass tag (TMT) approaches have become increasingly popular due to the capability to multiplex analysis of up to 18 complex matrices simultaneously [20]. TMT protocols enable samples to be pooled prior to nano liquid chromatography–tandem mass spectrometry (nLC-MS/MS), saving instrument time and reducing technical variations in the workflow.

However, the high cost of reagents, fixed number of samples, and sample preparation time and complexity combine to limit the utility of TMT protocols for the impromptu assessment of patient specimens in real-time and hence their broad uptake in the clinic. Label-free quantitation (LFQ) strategies provide quantitative phosphoproteomic data without the use of isotopic-tags, mainly through the direct inference of protein abundance using the measured intensity of detected peptides, or indirect inference based on the number of phosphopeptide-spectrum matches (PSMs) obtained for each protein [21]. LFQ protocols have the capacity to overcome some of the TMT-workflow limitations by reducing the complexity of sample preparation, saving both time and money on costly reagents. Additionally, there is no limit to the number of matrices that can be analyzed, enabling the comparison of larger sets of samples than when using label-based approaches. Such strategies therefore hold obvious appeal in the context of highly aggressive forms of cancer in which the design of appropriate treatment strategies is time-sensitive, and hence the ability to rapidly and reproducibly perform phosphoproteomic profiling is of critical importance [4]. However, label-free strategies have their own limitations, which include the inherent variability of individual sample preparation and loading, and the requisite number of replicates. Additionally, chromatographic conditions and the semirandom nature of data acquisition have also an impact in sample reproducibility [22]. The addition (spike-in) of known concentrations of standard heavy-labeled exogenous phosphopeptides for sample normalization, can help to overcome some of these limitations [17, 23], and therefore provides a strategy to normalize protein expression and phosphorylation abundance from different cancer specimens analyzed at any time.

The highly complex nature of cancer phosphoproteomes necessitates that sample preparation protocols are coupled with phosphopeptide enrichment and sample pre-fractionation prior to nLC-MS/MS analysis to achieve adequate phosphoproteome resolution. By interfacing phosphopeptide enrichment [12, 14, 24] with separation via High-field asymmetric waveform ion mobility spectrometry (FAIMS) [25, 26] prior to high-resolution mass spectrometry (MS), the collection of single-shot proteomic data is possible without the need to perform conventional two-dimensional liquid chromatography (2D-LC) approaches, and provides deep phosphoproteomic coverage to identify cancer-associated drug targets, in real-time. In seeking to combine the salient features of these analytical modalities, here we report the optimization of a new protocol that employs online phosphoproteome deconvolution in tandem with LFQ in the presence of internal control heavy-labeled standards. This protocol was optimized to identify kinases driving disease progression and therapy resistance in real-time. To determine the pre-clinical utility of this approach, pHASED was applied to isogenic FLT3-mutant AML cell lines resistant to the tyrosine kinase inhibitor sorafenib.

## Experimental procedures

### Cell culture

Murine hematopoietic progenitor FDC-P1 cells were stably transduced with either human *FLT3*-ITD, *FLT3*-ITD/D835V, or *FLT3*-ITD/D835Y by retroviral transduction [7], confirmed by standard Sanger sequencing (Additional file 1: Materials and Methods). FDC-P1 FLT3-transduced lines were maintained in standard culture conditions (5% CO_2_, 37 °C) in DMEM medium (Thermo Fisher Scientific) with the addition of 10% FBS, and 20 mM HEPES (*N*-2-hydroxyethylpiperazine-*N*′-2-ethanesulfonic acid). FLT3-mutant lines are factor-independent and were therefore maintained in growth factor free media. All cell lines were routinely confirmed to be free of mycoplasma contamination using a MycoAlert mycoplasma detection kit (Lonza; Basel, Switzerland).

### Sample preparation and protein extraction

Snap frozen transduced FDC-P1 cells expressing AML associated FLT3-mutations were lysed in 100 μL of ice-cold 0.1 M Na_2_CO_3_, pH 11.3 containing protease and phosphatase inhibitors (Sigma, cat. #P8340-5ML, and #4906837001 respectively), by sonication (2 × 20 s cycles, 100% output power) (as described [11, 24, 27]). Protein concentration was determined using a Bicinchoninic acid (BCA) protein estimation assay, as per manufacturer’s instructions (Thermo Fisher Scientific). Protein samples were then diluted in 6 M Urea/2 M Thiourea and reduced using 10 mM dithiothreitol (DTT) by incubation for 30 min at room temperature (RT). Reduced cysteine residues were then alkylated using 20 mM iodoacetamide by incubation for 30 min at RT in the dark. Enzymatic digestion was achieved using Trypsin/Lys-C mixture (Promega) at an enzyme-to-substrate ratio of 1:50 (w/w) and incubated for 3 h at RT. Triethylammonium bicarbonate (TEAB, 50 mM, pH 7.8) was then added to dilute urea concentration below 1 M, and samples were incubated overnight at RT. Lipid precipitation was performed using formic acid and trichloroacetic acid (TCA). Briefly, a final concentration of 2% formic acid was added to each sample, prior to centrifugation at 14,000×*g* for 10 min. Remaining lipopeptides were then precipitated with 20% (w/w) TCA and incubated on ice for at least 1 h prior to centrifugation. Pellets were washed with ice cold 0.01 M hydrochloric acid (HCl)/90% acetone and supernatants containing peptides were combined. Peptides were desalted using Oasis HLB solid phase extraction (SPE) cartridges and a VisiprepTM SPE Vacuum Manifold (12-port model; Sigma). The SPE cartridges were activated using 100% acetonitrile (ACN), equilibrated using 0.1% trifluoroacetic acid (TFA), and blocked with 33 µg of trypsin-digested bovine serum albumin (BSA) peptides prior to sample clean-up. Acidified samples (pH < 3) were loaded onto SPE cartridges with liquid passed through the solid phase dropwise using vacuum pressure. The cartridges were washed with 0.1% TFA followed by sequential elution of peptides using 60% ACN/0.1% TFA and 80% ACN/0.1% TFA. Eluted peptides were then resuspended in TEAB (50 mM, pH 8) and quantified using a Qubit 2.0 Fluorometer, as per manufacturer’s instructions (Thermo Fisher Scientific). A total of 200 µg of peptide per sample was utilized for TiO_2_ enrichment. Spike-in heavy-labeled phosphorylated peptides (Additional file 2: Table S1; including individually tyrosine, threonine or serine phosphorylated heavy-labeled spiketides, 8 fmol/200 μg of sample) were added as internal controls. Phosphopeptide enrichment was modified based on previous protocols [12, 14, 19]. In brief, each peptide sample was suspended in 80% ACN, 5% TFA, and 1 M glycolic acid (loading buffer). TiO_2_ beads were added at 0.6 mg per 100 µg peptide (w/w), and samples were mixed at RT for 15 min. The supernatant was incubated with half the amount of fresh TiO_2_ beads, and resultant supernatants containing non-phosphorylated peptides (non-modified = NM fraction) were removed and stored. The two sets of beads with bound phosphopeptides were pooled using 100 μL of loading buffer, followed by sequential washing with 80% ACN/1% TFA, and 10% ACN/0.1% TFA. Phosphopeptides were eluted with 28% ammonia hydroxide solution (1% v/v, pH 11.3) then passed through a C8 stage tip to remove residual beads [19]. Phosphopeptides were lyophilized completely prior to resuspension in 2% ACN/0.1% TFA for nLC-MS/MS analysis.

### Nanoflow liquid chromatography-tandem mass spectrometry (nLC-MS/MS)

Reverse phase nanoflow LC–MS/MS was performed using a Dionex Ultimate 3000RSLC nanoflow high-performance liquid chromatography system coupled with an Orbitrap Exploris 480 MS equipped with or without a front-end FAIMS Interface (Thermo Fisher Scientific) for pHASED or single-shot injection, respectively. Approximately 700 ng of phosphopeptide (single-shot injection, or per CV in pHASED) were loaded onto an Acclaim PepMap 100 C18 75 μm × 20 mm trap column for pre-concentration and online de-salting. The same chromatographic conditions were used in both experiments. Separation was achieved using an EASY-Spray PepMap C18 75 μm × 25 cm, employing a gradient of 0–35% solvent B (solvent A = 0.1% formic acid, solvent B = 90% ACN, 0.1% formic acid) at a flow rate of 250 nL/min over 75 min. For pHASED, the mass spectrometer was operated in positive mode with the FAIMS Pro interface. Four compensation voltages (CV; − 70, − 60, − 50, − 40) were individually run for each biological triplicate. Full MS/data dependent acquisition (DDA) was performed using the following parameters: Orbitrap mass analyzer set at a resolution of 60,000, to acquire full MS with an m/z range of 350–1200, incorporating a standard automatic gain control target of 1e^6^ and maximum injection time of 50 ms. The 20 most intense multiply charged precursors were selected for higher-energy HCD with a collisional energy of 30. MS/MS fragments were measured at an Orbitrap resolution of 15,000 incorporating a normalized automatic gain control target of 250% and a maximum injection time of 120 ms.

### Data processing and bioinformatic analysis

Data analysis was performed using Proteome Discoverer 2.4 (Thermo Fisher Scientific). Sequest HT was used to search against UniProt *Mus musculus* database (17,091 sequences, downloaded 25/04/2022) and *Homo sapiens* FLT3 FASTA file containing WT and mutant FLT3 sequences (3 sequences, downloaded 21/02/20). Database searching parameters included up to two missed cleavages, precursor mass tolerance set to 10 ppm and fragment mass tolerance of 0.02 Da. Cysteine carbamidomethylation was set as a fixed modification while dynamic modifications included phosphorylation (S, T, Y), and heavy-labeled 13C(6)15N(2) (K), and 13C(6)15N(4) (R) to identify spiked-in heavy-labeled phospho-spiketides. Interrogation of the database was performed to evaluate the false discovery rate (FDR) of peptide identification based on q-values estimated from the target-decoy search approach using Percolator. An FDR rate of 1% was set at the peptide level to filter out target peptide spectrum matches over the decoy-peptide spectrum matches. To account for variations in sample injection, reporter ion abundances were normalized to the spiked-in heavy-labeled phosphopeptides included as a FASTA file (Additional file 1: Fig. S1). For quantification and comparison, each ratio was transformed to log_2_ scale (log_2_ ratio).

### Experimental design and statistical rationale

Phosphoproteomic data analysis was performed using three FDC-P1 isogenic cell lines (n = 3 biological replicates). Four compensation voltages (CV; − 70, − 60, − 50, − 40) were individually analyzed for each biological replicate for pHASED. Differentially expressed phosphopeptides and phosphorylation sites were defined as those with a significant (*p* ≤ 0.05) log_2_ fold change ≥ 0.5 or ≤  − 0.5. Differences between sample groups were analyzed by unpaired Student’s t-tests or one-way ANOVA and considered significant when *p* ≤ 0.05. Graphical data was analyzed and prepared using Perseus (1.6.2.2), String (11.5), and GraphPad Prism (9.0.1). Results are presented as mean values ± SEM.

### Ingenuity pathway analysis

Ingenuity Pathway Analysis software (IPA; Qiagen) was used to analyze each phosphoproteomic dataset (as previously described [12, 14]). Canonical pathways, and upstream regulator analyses were generated and assessed based on *p*-value.

### Kinase-substrate enrichment analysis

Kinase-Substrate Enrichment Analysis (KSEA App, version 1.0) [28, 29] was used to analyze phosphorylated sites based on PhosphoSitePlus [30] kinase-substrate dataset, and a *p* ≤ 0.05 cut-off.

### Cell growth assay

Cell growth and viability were determined by trypan blue exclusion. Cells were seeded at 1e^5^ cells per mL. Cell number and viability were determined at 0 h, 24 h, and 48 h timepoints by trypan blue exclusion (n = 3 independent biological replicates). Doubling time was calculated using the following formula [27], and statistical significance determined via ordinary one-way ANOVA:

$$Doubling\;Time=\frac{\text{duration }*{\log}(2)}{\log\left(Final\;Concentration\right)-{\log}(Initial\;Concentration)}$$

### Cytotoxicity assays

Cell lines were treated with the FLT3 inhibitor sorafenib (Selleckchem) [31], and ATM inhibitor KU-60019 (Selleckchem) [32] either alone or in combination. Cells were seeded into 96 well plates at 2e^4^ cells per well, and viability following treatments was measured using Resazurin (excitation 544 nm, emission 590 nm; 0.6 mM Resazurin, 78 μM Methylene Blue, 1 mM potassium hexacyanoferrate (III), 1 mM potassium hexacyanoferrate (II) trihydrate (Sigma), dissolved in sterile phosphate buffered saline). Synergy of dose–response and combined effect of the two drugs were assessed using the method of Bliss independence model [33].

## Results

### pHASED provided improved phosphopeptide quantification compared to the LFQ workflow

**Fig. 1:**
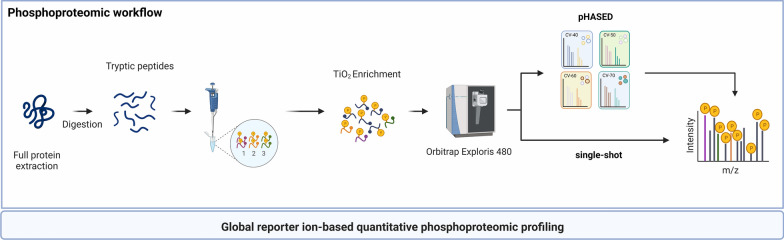
Overview of sample preparation and instrument workflow for quantitative phosphoproteomics using single-shot LFQ and pHASED. Proteins were extracted from target cell lines and digested into peptides. Two hundred micrograms of digested peptides were prepared for enrichment. In pHASED, known concentrations of spike-in heavy labeled phosphopeptides were added to each sample prior to phosphopeptide enrichment. For both single-shot LFQ and pHASED enriched phosphopeptides were then injected into an Orbitrap Exploris 480. In pHASED, nLC-MS/MS was coupled with a FAIMS interface using four different compensation voltages (CVs; − 70 V, − 60 V, − 50 V and − 40 V). Figure created with BioRender.com

Phosphopeptide enrichment using TiO_2_ [12, 13, 19, 24], coupled with LFQ using ‘pHASED’ described herein, employs heavy-labeled internal phospho-spiketides and FAIMS interface [25, 26], to increase phosphoproteome deconvolution and coverage for the analysis of samples in real-time (Fig. 1). To simulate the limited amount of starting material that is often provided by clinical teams following a cancer patient’s biopsy, resection, or bone marrow aspiration, we used a maximum of 200 µg of starting material. We performed initial comparison of our optimized pHASED protocol with traditional single-shot LFQ phosphoproteomics using three isogenic cell line models of FLT3-mutant AML in biological triplicate (Table 1; n = 9 samples/experiment). The sample preparation in pHASED includes the addition of spike-in of heavy-labeled phospho-spiketides of known concentration to normalize sample injection and phosphopeptide quantitation (Additional file 1: Fig. S1). To increase phosphoproteome coverage, we employed online deconvolution using FAIMS interface employing external stepping of four different compensation voltages (CV; − 70, − 60, − 50, − 40) over a 75 min gradient. Individual sample injection per CV provided more in-depth coverage (Fig. 1).

**Table 1:** Isogenic cellular models of FLT3 mutant AML analyzed by pHASED or single-shot LFQ protocols in biological triplicate (n = 9/technique, 18 total)

Cell line	Species	Biological replicates	Samples	Model of AML stage	Sorafenib sensitivity
FDC-P1	Mouse	3	FLT3-ITD	Diagnosis	Sensitive
FDC-P1	Mouse	3	FLT3-ITD/D835V	Relapse	Resistant
FDC-P1	Mouse	3	FLT3-ITD/D835Y	Relapse	Resistant

**Fig. 2:**
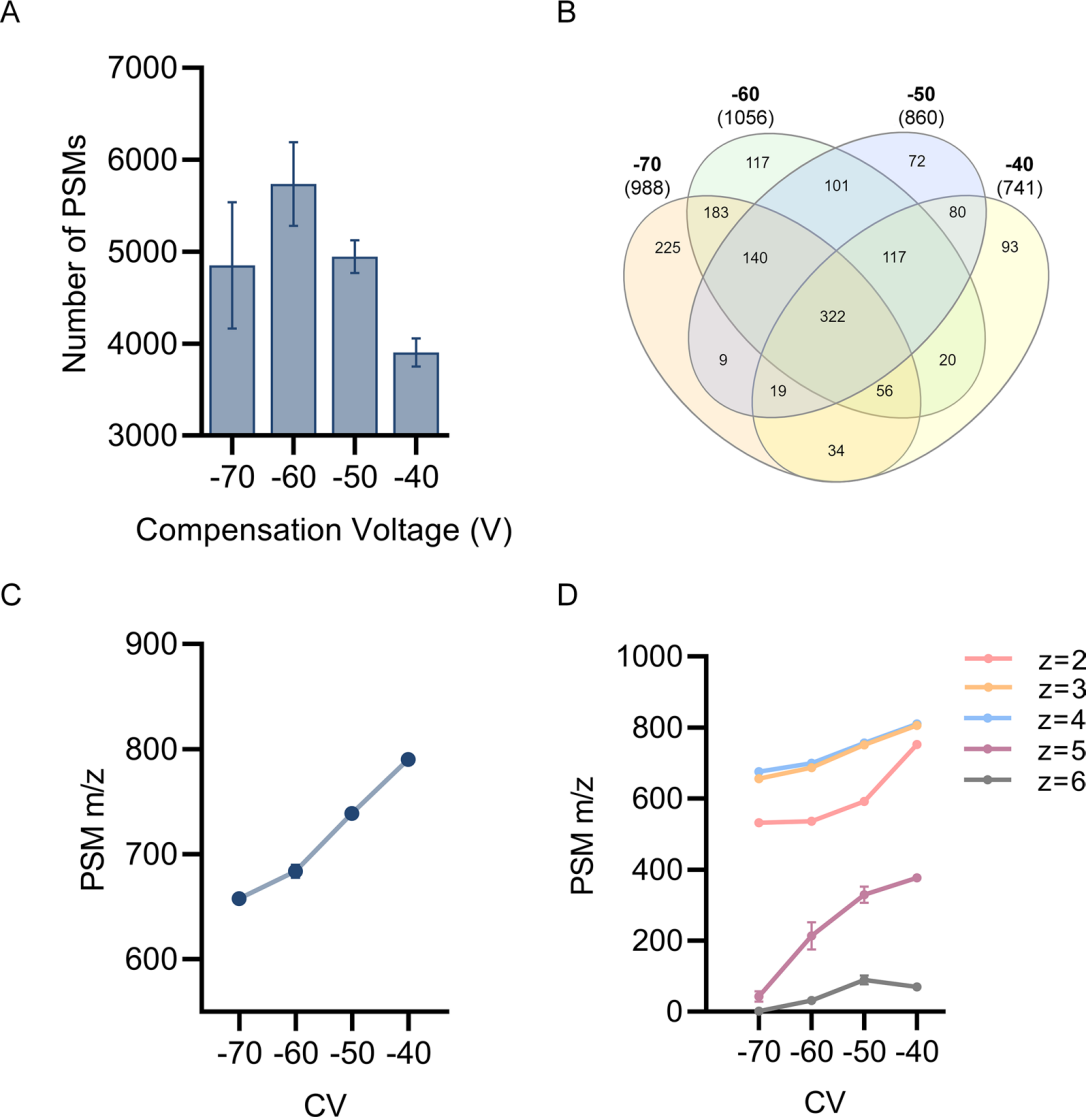
Acquisition profile of phosphopeptide-spectrum matches (PSMs) resulting from single-shot LFQ compared to pHASED. **A** Number of PSMs identified in each CV. **B** Venn distribution of unique phosphoprotein accessions identified in CVs − 70 V, − 60 V, − 50 V and − 40 V shows overall coverage of common and unique acquisitions detected in each CV. **C** Average m/z of all PSM features acquired in each CV. **D** Average m/z of PSM features for charge states acquired in each CV

pHASED analysis of isogenic AML cell lines identified increased numbers of PSMs at lower CVs (Fig. 2A, B), with the greatest number of unique PSMs identified at − 60 V (Fig. 2A, B*,* Additional file 2: Table S2) [34]. Similar PSM distributions were seen across CVs as previously reported for studies employing FAIMS to characterize the proteome [26] and phosphoproteome [25] (Fig. 2A, B). Analysis of PSMs identified that lower CVs tended to harbor lower m/z (Fig. 2C), across charge states (Fig. 2D).

**Fig. 3:**
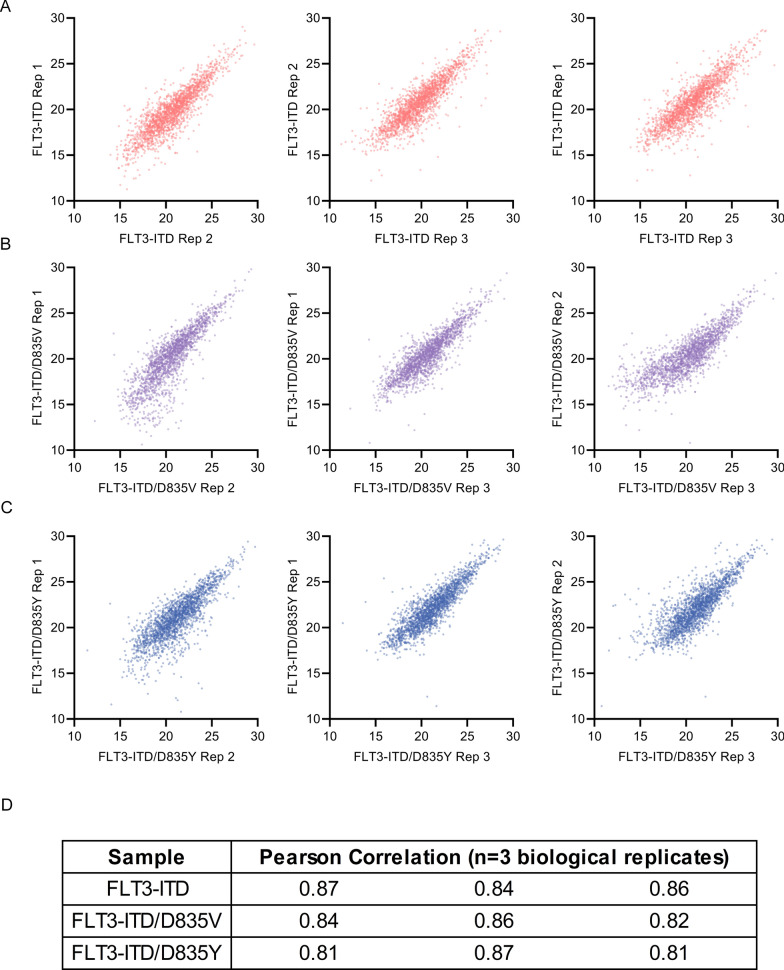
Quantification reproducibility between biological replicates. Pearson correlation profiles for biological replicates (n = 3) of the three isogenic models of acute myeloid leukemia. **A** FLT3-ITD, **B** FLT3-ITD/D835V, and **C** FLT3-ITD/D835Y analyzed by pHASED. **D** Comparison of all three correlation scores achieved by FLT3-mutant isogenic cell lines. Correlation was performed using normalized abundances in Perseus

To examine the reproducibility of the phosphopeptide quantification achieved using pHASED we performed Pearson Correlation analysis of the biological replicates (n = 3) across each cell line (n = 3) (Fig. 3). Correlation was performed by plotting normalized phosphopeptide abundances from each biological replicate per sample in a correlation matrix. These analyses confirm the fidelity and reproducibility of pHASED across samples and biological replicates (Fig. 3A–D) (average r = 0.84 ± 0.03).

### pHASED provided in-depth phosphoproteome coverage

**Fig. 4:**
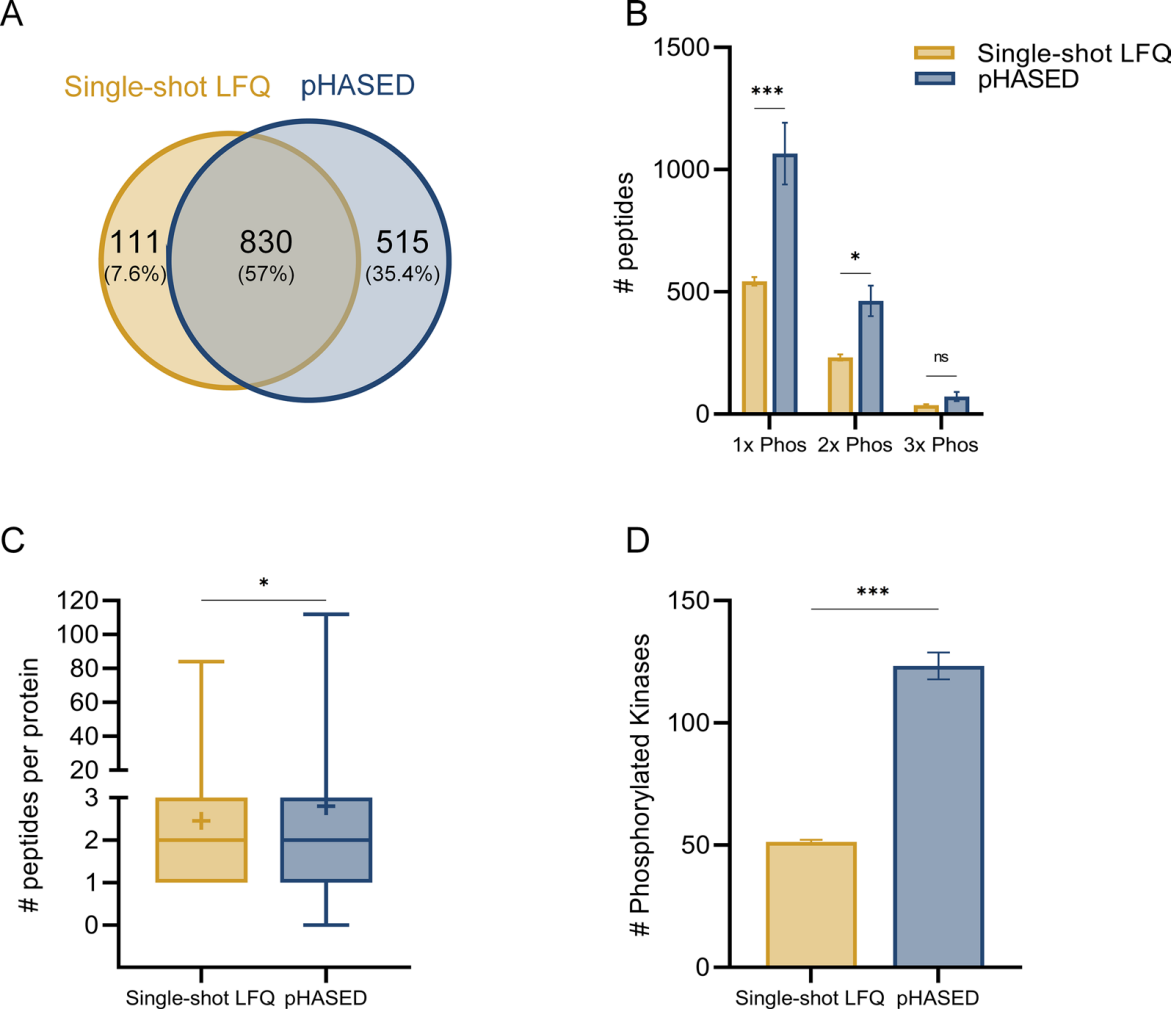
Analysis of phosphoproteome and phosphopeptide characteristics identified using single-shot LFQ compared with pHASED. **A** Overlap of phosphoprotein accessions comparing single-shot LFQ and pHASED experiments. **B** Number of single and multi-phosphorylated peptides identified in each experiment comparing single-shot LFQ and pHASED (statistical significance was determined via ordinary two-way ANOVA). **C** Number of phosphopeptides per protein identified in single-shot LFQ and pHASED experiments (unpaired t-test). **D** Number of high confidence phosphorylated master protein kinases identified in each single-shot LFQ and pHASED experiment (unpaired t-test). Statistical threshold: *p < 0.05, **p < 0.01 and ***p < 0.001

The single-shot LFQ approach identified 941 unique phosphoproteins and 2467 unique phosphopeptides (FDR 1%), whereas, pHASED identified 1345 unique phosphoproteins and 3877 unique phosphopeptides (FDR 1%). Comparison between identified unique phosphoproteins across all datasets showed a 57% overlap between pHASED and single-shot LFQ (Fig. 4A, Additional file 2: Table S5). Overall, phosphopeptide enrichment for single-shot LFQ was 91%, and 93% for pHASED. Comparatively, pHASED identified more unique phosphorylation sites compared to the single-shot LFQ approach (4725 versus 3784, respectively) (Additional file 2: Tables S3, S4), including more single, and doubly-phosphorylated peptides (pHASED = 1066 singly-, 463 doubly-, and 71.67 triply-phosphorylated peptides; single-shot LFQ = 543 singly-, 232 doubly-, and 36.33 triply-phosphorylated peptides, all FDR 1%, p ≤ 0.05) (Fig. 4B). Notably, pHASED also provide increased phosphoproteomic coverage, identifying significantly more phosphopeptides per protein compared to LFQ (*p* = 0.03) (Fig. 4C).

### pHASED identified relevant therapeutic drug targets in drug resistant AML

**Fig. 5:**
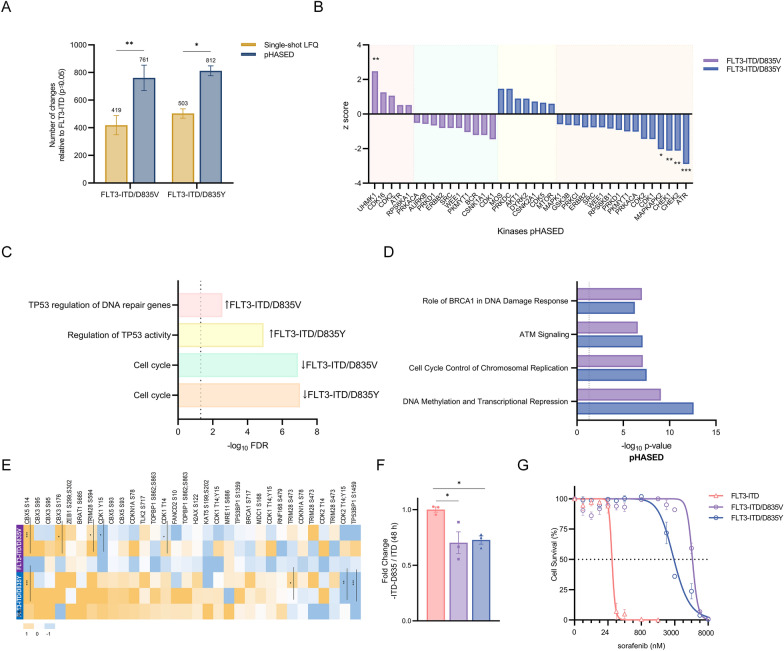
Analysis of FLT3 resistance via pHASED. **A** Number of differentially phosphorylated peptides (log_2_ ± 0.5, *p* ≤ 0.05) in double mutants in comparison to FLT3-ITD cell using single-shot LFQ and pHASED datasets. **B** Kinase substrate enrichment analysis (KSEA) profile of resistant cell lines compared to FLT3-ITD. Z score indicates predicted kinase activity, with a positive value predictive of kinase activation and a negative value predictive of kinase inhibition. **C** Reactome enrichment profile of kinases identified by KSEA as differentially regulated (log_2_ ± 0.5) in resistant cell lines compared to FLT3-ITD cells. Top molecular functions and/or signaling identified by Reactome have been assigned to each cluster if significantly over-represented by kinases. **D** Common canonical pathways identified by Ingenuity Pathway analysis (IPA) of phosphorylated changes in resistant cell lines compared with FLT3-ITD. **E** Phosphorylation profile of ATM signaling substrates in resistant cell lines compared with FLT3-ITD. Yellow indicates increased phosphorylation, whereas blue represents decreased phosphorylation. **F** Proliferation rate after 48 h comparing FLT3-ITD/D835 to FLT3-ITD (ratio of triplicates ± SE are shown, and statistical significance was determined via ordinary one-way ANOVA, *p < 0.05). **G** Confirmation of differential response to treatment with tyrosine kinase inhibitor (TKI) sorafenib. Cell viability was assessed by Resazurin assay at 48 h treatment (statistical significance calculated using one-way ANOVA, *p < 0.05, **p < 0.01 and ***p < 0.001)

Importantly, pHASED identified 2.4× more phosphoproteins (1% FDR) with predicted kinase activity than single-shot LFQ (mean = 51.33 for LFQ; 123.3 for pHASED; *p* = 0.0002) (Fig. 4D, Additional file 2: Tables S6, S7). Like many other cancers, dysregulated kinase activity is a main feature driving AML oncogenic signaling, with the most common mutations seen at diagnosis in the receptor tyrosine kinase FLT3 (20–35% patients) [35, 36]. To investigate the utility of pHASED for the identification of kinases, and hence drug targets outside of the mutant FLT3-receptor seen in these cells (FLT3-ITD, resistant FLT3-ITD/D835V, and resistant FLT3-ITD/D835Y), Kinase-Substrate Enrichment Analysis (KSEA) and Ingenuity Pathway Analysis (IPA) was employed using the phosphorylation profile identified by both methods (Additional file 2: Tables S9–S12, Fig. 5). FLT3-ITD mutations are seen in approximately 27% of AML patients at diagnosis and are associated with a high risk of relapse [3]. Resistance to commonly used tyrosine kinase inhibitors (TKIs) including sorafenib (Additional file 2: Table S8) in FLT3-ITD + AML patients, commonly occurs following the acquisition of a secondary point mutation at aspartic acid 835 in the kinase domain of FLT3-ITD (FLT3-ITD/D835V and FLT3-ITD/D835Y, henceforth referred to as “double mutant”) [37, 38].

pHASED identified significantly more proteins harboring altered phosphorylation patterns (log_2_ ± 0.5; *p* ≤ 0.05) in double mutant cells compared to FLT3-ITD cells (Fig. 5A; *p* ≤ 0.01). As a result, KSEA and IPA (log_2_ ± 0.5) provided a more robust readout of oncogenic signaling, including greater number of differentially regulated kinases (average = 41 pHASED; 22 single-shot LFQ) (Additional file 2: Tables S9, S10), and canonical pathways (average = 77 pHASED; 27 single-shot LFQ) (Additional file 2: Table S11, S12). Taken together, pHASED is more effective at identifying clinically relevant drug targets to aid in the design of treatment strategies for cancer patients.

To determine whether pHASED could identify a treatment regimen to act as a salvage strategy for sorafenib resistant AML, significant changes in phosphorylation in FLT3-ITD/D835 double mutant cells were compared to FLT3-ITD cell lines (log_2_ ± 0.5) and each FLT3-ITD/D835 individual dataset analyzed using KSEA. Oncogenic signaling pathways enriched with kinases were then explored using Reactome (Fig. 5B, C). In resistant cells, kinases associated with p53 activity and the regulation of DNA repair were upregulated (*p* < 0.001), including Casein kinase 2 alpha 1 (CSNK2A1) [39, 40], Dual specificity tyrosine-phosphorylation-regulated kinase 2 (DYRK2) [41], Cyclin-dependent kinase 5 (CDK5) [42], Serine/threonine-protein kinase mTOR (mTOR) [43], RAC-alpha serine/threonine-protein kinase; (AKT1) [44], and DNA-dependent protein kinase (DNA-PK) [45]. For each comparison, Reactome also identified ‘cell cycle’ as the biological process overrepresented by kinases with decreased activity (*p* < 0.001), including Serine/threonine-protein kinases 1/2 (CHEK1/2) [46], Aurora kinase B (AURKB) [47], CDK1 [48], Wee1-like protein kinase (WEE1) [49], and Membrane-associated tyrosine- and threonine-specific cdc2-inhibitory kinase (PKMYT1) [49]. Cell cycle regulator CDK2 [48], and DNA damage sensor Ataxia telangiectasia and rad3 related (ATR) [50] kinases were predicted as activated in FLT3-ITD/D835V cells, but inactivated in FLT3-ITD/D835Y (Fig. 5B, Additional file 2: Table S13).

### pHASED identified DNA damage and repair pathways associated with sorafenib resistance in FLT3-mutant AML

In accordance with KSEA predictions, IPA of the phosphoproteomes of resistant cells (Fig. 5D, Additional file 2: Table S12) identified cell cycle regulation (Cell cycle control of Chromosomal Replication, *p* < 0.001), and DNA damage and repair ATM signaling (*p* < 0.001) as the most significantly enriched pathways. Both mutants were also enriched for FLT3, and AML associated signaling pathways such as ERK/MAPK, JAK/STAT, mTOR, and PI3K/AKT, although with less power (Additional file 2: Table S12).

The Serine/threonine protein kinase (ATM) regulates response to DNA damage caused by double-strand breaks (DSBs) [14]. ATM is a member of the phosphoinositide 3-kinase (PI3K)-related protein kinase (PIKK) family, and signals through DNA damage response kinases ATR, DNA-PKcs and Nonsense Mediated MRNA Decay Associated PI3K Related Kinase (SMG1) [51, 52]. One potent mechanism of increased DSBs is via the excess production of reactive oxygen species (ROS) [53, 54]. Increased ROS production by the NADPH oxidase (NOX) family of enzymes in acute leukemias, particularly FLT3-ITD AML, has been increasingly studied over the last few years, and highlights that elevated ROS is a mechanism conferring survival advantages in FLT3-mutant AML [53–56]. Given ATM signaling was predicted to be one of the top ranked canonical pathways driving the DNA damage repair and response pathways in resistant cells (Fig. 5D), we chose to analyze this pathway to test the biological utility of the phosphoproteomic analysis generated via pHASED in FLT3-ITD/D835V and FLT3-ITD/D835Y mutant cells.

Analysis of ATM signaling including downstream DSB repair phosphoproteins (Fig. 5E) in resistant models revealed divergent phosphorylation profiles. Compared with FLT3-ITD cells, in FLT3-ITD/D835V cells, pHASED identified significantly increased phosphorylation of transcriptional regulators Chromobox protein homolog 3 (CBX3; S176) (log_2_ 1.73, *p* = 0.02), and CBX5 (S14) (log_2_ 6.64, *p* < 0.001) (Fig. 5E), as well as the ATM-regulated Transcription intermediary factor 1-beta (TRIM28; S594) (log_2_ 2.07, *p* = 0.02); whereas phosphorylation of cell cycle regulator CDK1 (T14;Y15) (log_2_ − 1.56, *p* = 0.01) was decreased. In FLT3-ITD/D835Y mutant cells, pHASED identified significantly increased phosphorylation of CBX5 (S14) (log_2_ 6.64, *p* < 0.001), and decreased phosphorylation of TRIM28 (S473) (log_2_ − 0.62, *p* = 0.03), CDK2 (T14;Y15) (log_2_ − 2.59, *p* = 0.003), and TP53-binding protein 1 (TP53BP1; S1459) (log_2_ − 6.64, *p* < 0.001) (Fig. 5E).

To determine whether the KSEA (Fig. 5B, C) and IPA (Fig. 5D) that predicted deregulation of cell cycle signaling in the resistant cells influenced the growth rate of our models, we profiled the proliferation capacity of our models, identifying a significantly reduced proliferation rate − 1.4× in the double mutants compared to FLT3-ITD cells (FLT3-ITD/D835V = − 1.42 fold-decrease, *p* = 0.01; FLT3-ITD/D835Y = − 1.38 fold-decrease, *p* = 0.02) (Fig. 5F).

### Combined inhibition of ATM and FLT3 showed synergistic cell death in sorafenib-resistant cell lines

**Fig. 6:**
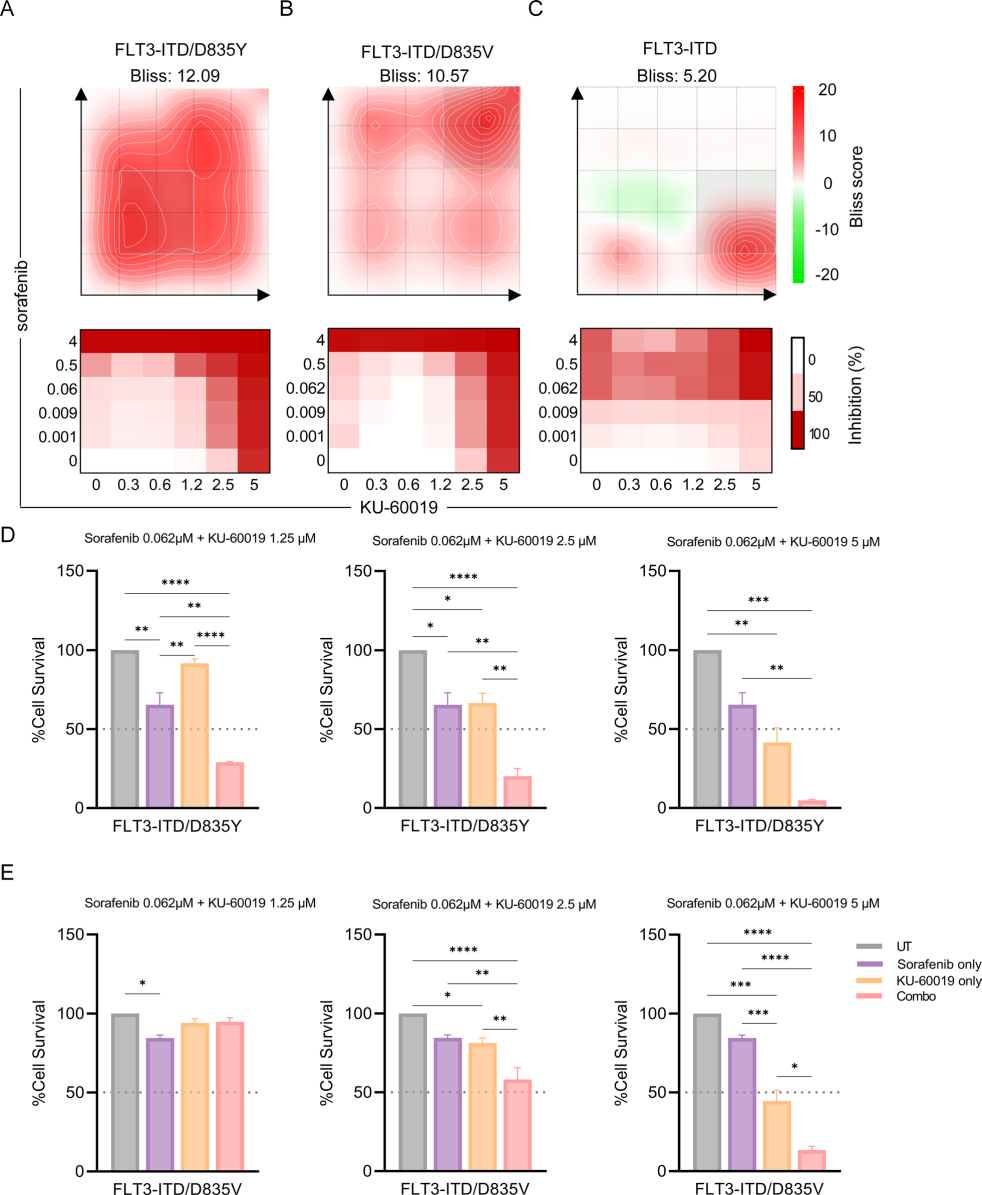
AML cell line model sensitivity to ATM inhibition in combination with FLT3 inhibitor. Bliss synergy analysis of combined sensitivity of AML cell line models to sorafenib and KU-60019, **A** FLT3-ITD/D835Y, **B** FLT3-ITD/D835V, and **C** FLT3-ITD (< 0 = antagonistic, > 0 < 10 = additive, > 10 = synergistic). Cell survival comparison of **D** FLT3-ITD/D835Y, and **E** FLT3-ITD/D835V cell lines at 62 nM sorafenib in combination with 1.25 µM, 2.5 µM and 5 µM KU-60019 (n = 3 independent replicates, statistical significance calculated via one-way ANOVA *p < 0.05, **p < 0.01 and ***p < 0.001)

Confirming the utility of our models, FLT3-ITD/D835V and FLT3-ITD/D835Y mutants (Fig. 5G) presented, on average, a 48-fold increased resistance to sorafenib compared with FLT3-ITD cell lines (*p* ≤ 0.01) (n = 3 independent biological replicates) (Additional file 2: Table S14). Based on the predictions of ATM signaling activation in sorafenib resistant models, combination cytotoxicity analysis was performed using the ATM inhibitor KU-60019 [32] in combination with the FLT3 inhibitor sorafenib [31] (Fig. 6). The combined inhibition of ATM and FLT3 signaling was highly synergistic, particularly in cells harboring the sorafenib resistant causing mutation FLT3-ITD/D835Y (Fig. 6A). FLT3-ITD/D835Y mutant cells showed increased sensitivity to the combination, resensitizing cells to sorafenib (Fig. 6A, Bliss synergy analysis score 12.09 at 0.062 µM sorafenib, 1.25 µM KU-60019); whereas FLT3-ITD/D835V mutant cells showed synergy at higher doses (Fig. 6B, Bliss score 10.57 at 0.062 µM sorafenib, 2.5 µM KU-60019). The combined inhibition of ATM and FLT3 signaling was only additive in FLT3-ITD mutant cells (Bliss score 5.20 at 0.001 µM sorafenib, 5 µM KU-60019) with these cells highly sensitive to sorafenib alone (Figs. 5G, 6C) (Additional file 2: Table S15). To test the in vitro preclinical benefits using physiologically relevant concentrations of sorafenib, cell survival comparisons were performed at 0.062 µM sorafenib. Again, these data confirmed the increased synergistic effects of combined ATM and FLT3 inhibition in FLT3-ITD/D835Y mutant cells (Fig. 6D) compared with FLT3-ITD/D835V mutant cells (Fig. 6E). Together, these results confirm that the inhibition of ATM and hence DNA repair plays a role in the resensitization of FLT3-ITD/D835 mutant resistant cells to sorafenib. Further, this validates the pHASED phosphoproteomic prediction of the importance of ATM signaling downstream of FLT3-ITD/D835 mutations.

## Discussion

Proteomics and phosphoproteomics have been acknowledged as being among the most effective strategies to predict drug sensitivities [1, 57]. However, we are yet to establish phosphoproteomic profiling in the clinical setting, or even to provide an additional resource to genomically predicted therapeutic strategies; the establishment of which would represent a pivotal advance in precision-medicine treatment regimens. Clinical phosphoproteomic profiling has enormous potential to identify treatment targets for cancers that harbor untargetable mutations like seen in uniformly lethal pediatric high-grade gliomas [1, 4, 58], or that are invisible to genomics approaches like those described herein, or phosphoproteomes to be used as an indicator of prognosis in the de novo and refractory settings, in real-time. Indeed, the optimization of pHASED reported herein, goes some way to moving phosphoproteomics from the discovery laboratory to that of the well-equipped pathologist. The reduced sample preparation complexity coupled with the deep phosphoproteomic coverage possible with pHASED, provides users with the capacity to prepare, fractionate, and sequence the phosphoproteome of any biological system in less than a week. Due to the combination of accurate LFQ and online deconvolution using FAIMS, pHASED maintained deep phosphoproteomic coverage without the need for offline 2D-LC techniques. Compared to single-shot analysis, pHASED increased LC–MS/MS run time by 1.8 days (45 h for pHASED; 11.25 h for LFQ); a limitation compensated by the increased phosphoproteomic depth of coverage, and the identification of a more targetable kinases. Furthermore, in our attempt to establish a robust, reproducible platform for the prediction of novel therapeutic targets in clinically relevant timeframes for AML patients resistant to therapies, we also used unnormalized pHASED phosphoproteomic data, as the phosphorylation of key DNA repair pathway proteins identified herein were not influenced by the abundance of the respective protein.

FAIMS was initially and elegantly optimized to provide single-shot LC–MS/MS results that compared favorably with 2D-LC fractionation experiments [26]. Specifically, FAIMS was previously reported in the context of analyzing the global phosphoproteome of a cell line. MS/MS analyses using FAIMS, identified 15% more unique phosphosites compared to single-shot analysis without FAIMS. Accordingly, our pHASED workflow using isogenic models of AML, identified a 25% increase in the number of unique phosphosites identified compared to single-shot analysis. Furthermore, pHASED also identified 50% more multiphosphorylated peptides compared to single-shot analysis without FAIMS, similar to the increase identified in previous studies using FAIMS, including the analysis of different sample types [25].

Analyzing the signaling pathways identified by both MS approaches provided molecular insights to help dissect therapeutic vulnerabilities. Indeed, pHASED identified considerably more deregulated kinases and canonical pathways compared to single-shot analysis, with IPA predicting increased ATM signaling in both FLT3-ITD/D835V and FLT3-ITD/D835Y double mutant cell lines compared to FLT3-ITD mutations alone. ATM plays a functional role in the cellular response to DNA DSBs. Here it protects the cell against genotoxic stress, but, in cancer cells, helps to drive resistance to anticancer therapies thus favoring leukemic growth and survival [59]. Therefore, it is unsurprising that ATM signaling may play a role in resistance to sorafenib in cells harboring double mutant FLT3-ITD/D835. However, sorafenib is not only a potent inhibitor of wt-FLT3 and FLT3-ITD, but also inhibits other receptor tyrosine kinases including VEGFR, PDGFR, KIT and RET, as well as downstream serine/threonine kinases including RAF/MEK/ERK [31]. In glioma cells, ATM inhibitors increased radiotherapy sensitivity [32, 60, 61], with ATM signaling through the RAF/MEK/ERK pathway critical for radiation-induced ATM activation, suggestive of a regulatory feedback loop between ERK and ATM [62]. Indeed, this feedback loop may help to explain the 48-fold increase in IC_50_ seen between the cell types. Sorafenib dose-dependently induced the generation of ROS in tumor cells in vitro and in vivo [63], and hence it is highly possible that in FLT3-ITD/D835 double mutants, RAF/MEK/ERK signaling through ATM helps to maintain proliferation and promote DNA repair, even under situations of genotoxic stress induced by high dose sorafenib.

pHASED identified more unique phosphorylation changes in ATM substrates in FLT3-ITD/D835Y cells compared to FLT3-ITD/D835V cells (log_2_ ≥ 0.5) (Additional file 2: Table S16). Combination cytotoxicity assays revealed significantly increased synergy between sorafenib and the ATM inhibitor KU-60019 at physiologically relevant doses (most strikingly and unsurprisingly in FLT3-ITD/D835Y cells) thus providing a treatment paradigm for patients harboring sorafenib resistance. It is interesting to note that the double mutant FLT3-ITD/D835Y showed decreased ATR activity by KSEA, whereas FLT3-ITD/D835V cells showed predictions of ATR activation. The difference in sensitivity may result from the cross-regulation seen between ATM and ATR at different stages of DDR [64]. The increased phosphosite coverage resulting from pHASED analyzes provides a more accurate indication of the regulation of the ATM signaling pathway, and hence highlights mechanisms promoting resistance to sorafenib [13]; information that can be exploited to tailor effective preclinical treatment strategies.

Although pHASED may afford the opportunity to perform an unrestricted number of analyzes (of benefit in the clinical setting where cancer diagnosis never follows a predictable schedule), there remains important questions about how phosphoproteomics would be practically implemented as a clinical decision-making tool. For example, consideration needs to be given to sample processing time and the methods of patient sample collection; the phosphoproteome of leukemic blasts isolated from the bone marrow will differ from those sequenced from leukemic blasts isolated from peripheral blood. Additionally, the steps taken to enrich leukemic blasts following bone marrow trephine biopsy or phlebotomy are to be considered as alterations in signaling pathway activity can be influenced simply by the culture media used, or even the type of blood tube used at the time of sample collection [27], necessitating optimization and standardization of workflows. Importantly, for phosphoproteomics to aid in the selection of treatments for cancer patients, the assessment of which pathways should be targeted and by which drugs needs to be evaluated under clinical trial conditions, like those testing whole genome sequencing (WGS) and RNA sequencing (RNAseq) strategies, in order to ensure robust recommendations can be made based on phosphoproteomic data generated via pHASED [65].

In summary, the data generated here provides an optimized method for LFQ and high-throughput phosphoproteomic data generation from limited amounts of starting material that maintains deep phosphoproteomic coverage without the need for complex 2D-LC strategies. pHASED provides the flexibility to analyze samples as they present and is not limited by the number of analyzes that can be performed. Reduced time and sample preparation complexity, and the optimization of online phosphoproteome deconvolution using a stepped CV FAIMS interface, provides an accurate tool for the reproducible characterization of complex cancer cell phosphoproteomes in less than a week. Moreover, pHASED successfully identified novel drug targets and potential therapeutic strategies to treat AML models resistant to therapies used in the clinic. We hope this optimized technology will help in the rapid characterization of other highly aggressive forms of cancer, an important step towards improving treatment outcomes for cancer sufferers.

## Supplementary Information

**Additional file 1.** Additional materials and methods. **Figure S1.** Standard spike-in control abundances (n = 3 biological replicates). Abundances for heavy-labeled spike-in peptides phosphorylated at tyrosine (Y), threonine (T), and serine (S) residues for FLT3-mutant cell lines. **A** Raw and **B** normalized peptide abundances of spike-in controls according to different FLT3 cell lines. Total SBDS **C** raw and **D** normalized protein abundances for FLT3-ITD, FLT3-ITD/D835V, and FLT3-ITD/D835Y cell lines.

**Additional file 3: Table S1.** SBDS heavy-labeled phosphorylated peptide standards. **Table S2.** Common and unique phosphoproteins identified across all four CVs based on PSM acquisition. **Table S3.** High confidence modification sites identified in LFQ (p < 0.01). **Table S4.** High confidence modification sites identified in pHASED (p < 0.01). **Table S5.** Unique and common phosphoproteins identified in LFQ and pHASED datasets. **Table S6.** Phosphorylated master protein kinases identified in LFQ dataset (p < 0.01). **Table S7.** Phosphorylated master protein kinases identified in pHASED dataset (p < 0.01). **Table S8.** FLT3-D835 mutations associated with resistance to tyrosine kinase FLT3 inhibitors. **Table S9.** Kinase-Substrate analysis of LFQ dataset for resistant cells in comparison to FLT3-ITD (log2 fold change ± 0.5). **Table S10.** Kinase-Substrate analysis of pHASED dataset for resistant cells in comparison to FLT3-ITD (log2 fold change ± 0.5). **Table S11.** Canonical pathways identified as significantly associated with LFQ dataset for resistant cells in comparison to FLT3-ITD. **Table S12.** Canonical pathways identified as significantly associated with pHASED dataset for resistant cells in comparison to FLT3-ITD. **Table S13.** Kinase activity inferred by KSEA analysis of phosphorylation changes in pHASED dataset (log2 ± 0.5, p ≤ 0.05) for resistant cells in comparison to FLT3-ITD. **Table S14.** Mutation-specific response to sorafenib. IC50 compared to FLT3-ITD. **Table S15.** Bliss Synergy scores for sorafenib in combination with KU-60019 at different doses. **Table S16.** Unique ATM substrates identified with increased phosphorylation (log2 ≥ 0.5) in pHASED dataset for resistant cells in comparison to FLT3-ITD. **Table S17.** Vector mutations in FLT3 gene.

## Data Availability

The mass spectrometry proteomics data have been deposited to the ProteomeXchange Consortium (http://proteomecentral.proteomexchange.org) via the PRIDE partner repository [66] with the dataset identifier. Project name: Phospho heavy-labeled-spiketide FAIMS stepped-CV DDA (pHASED) provides real-time phosphoproteomics data to aid in cancer drug selection Project accession: PXD037227. Project DOI: not applicable. Reviewer account details—Username: reviewer_pxd037227@ebi.ac.uk. Password: jPljYUtp.

## Abbreviations

2D-LC: Two-dimensional liquid chromatography
ACN: Acetonitrile
AKT1: RAC-alpha serine/threonine-protein kinase
AML: Acute myeloid leukemia
ATM: Ataxia-telangiectasia mutated
ATR: Ataxia telangiectasia and rad3 related
AURKB: Aurora kinase B
BCA: Bicinchoninic acid
BSA: Trypsin-digested bovine serum albumin
CBX3: Chromobox protein homolog 3
CBX5: Chromobox protein homolog 5
CDK1: Cyclin-dependent kinase 1
CDK2: Cyclin-dependent kinase 2
CDK5: Cyclin-dependent kinase 5
CHEK1/2: Serine/threonine-protein kinases 1/2
CSNK2A: Casein kinase 2 alpha 1
CV: Compensation voltages
DDA: Data dependent acquisition
DSBs: Double-strand breaks
DTT: Dithiothreitol
DYRK2: Dual specificity tyrosine-phosphorylation-regulated kinase 2
FAIMS: High-field asymmetric waveform ion mobility spectrometry
FDR: False discovery rate
HCl: Hydrochloric acid
HPLC: High-pressure liquid chromatographic
IPA: Ingenuity Pathway Analysis
ITD: Internal tandem duplications
KSEA: Kinase-Substrate Enrichment Analysis
LFQ: Label-free quantitation
MS: Mass spectrometry
mTOR: Serine/threonine-protein kinase mTOR
nLC-MS/MS: Nanoflow liquid chromatography–tandem mass spectrometry
NM: Non-modified
NOX: NADPH oxidase
pHASED: Phospho heavy-labeled-spiketide FAIMS stepped-CV DDA
PI3K: Phosphoinositide 3-kinase
PKMYT1: Membrane-associated tyrosine- and threonine-specific cdc2-inhibitory kinase
PRKDC; DNA-PK: DNA-dependent protein kinase
PSMs: Phosphopeptide-spectrum matches
RNAseq: RNA sequencing
ROS: Reactive oxygen species
RT: Room temperature
SMG1: Nonsense Mediated MRNA Decay Associated PI3K Related Kinase
SPE: Solid phase extraction
TCA: Trichloroacetic acid
TEAB: Triethylammonium bicarbonate
TFA: Trifluoroacetic acid
TKIs: Tyrosine kinase inhibitors
TMT: Tandem mass tag
TP53BP1: TP53-binding protein 1
TRIM28: ATM-regulated Transcription intermediary factor 1-beta
WEE1: Wee1-like protein kinase
WGS: Whole genome sequencing
