# Correction to: Phospho-heavy-labeled-spiketide FAIMS stepped-CV DDA (pHASED) provides real-time phosphoproteomics data to aid in cancer drug selection

**DOI:** 10.1186/s12014-023-09406-z

**Published:** 2023-04-08

**Authors:** Dilana E. Staudt, Heather C. Murray, David A. Skerrett-Byrne, Nathan D. Smith, M. Fairuz B. Jamaluddin, Richard G. S. Kahl, Ryan J. Duchatel, Zacary P. Germon, Tabitha McLachlan, Evangeline R. Jackson, Izac J. Findlay, Padraic S. Kearney, Abdul Mannan, Holly P. McEwen, Alicia M. Douglas, Brett Nixon, Nicole M. Verrills, Matthew D. Dun

**Affiliations:** 1 School of Biomedical Sciences and Pharmacy, College of Health, Medicine and Wellbeing, University of Newcastle, Callaghan, NSW 2308 Australia; 2 Precision Medicine Research Program, Hunter Medical Research Institute, New Lambton Heights, NSW 2305 Australia; 3 School of Environmental and Life Sciences, College of Engineering, Science and Environment, University of Newcastle, Callaghan, NSW 2308 Australia; 4 Infertility and Reproduction Research Program, Hunter Medical Research Institute, New Lambton Heights, NSW 2305 Australia; 5 Analytical and Biomolecular Research Facility (ABRF), Research Services, University of Newcastle, Callaghan, NSW 2308 Australia

**Correction to: Clinical Proteomics (2022) 19:48**

10.1186/s12014-022-09385-7

Unfortunately, in the original publication of the article, the following errors were identified after online publication of the article.

The Additional file 2 was published with only one table (Table S14), whereas the remaining Tables S1-S17 were omitted. This error was caused due to typesetting mistake.

In Abstract, line 11, the text that reads as “phospho-heavy-labeled-spiketide FAIMS Stepped-CV DDA (pHASED)” should read as “hospho-heavy-labeled-spiketide FAIMS stepped-CV DDA (pHASED)”.

The original article has been corrected.

## Electronic supplementary material

**Additional file 2**: **Table S1**. SBDS heavy-labeled phosphorylated peptide standards. **Table S2**. Common and unique phosphoproteins identifed across all four CVs based on PSM acquisition. **Table S3**. High confdence modifcation sites identifed in LFQ (p < 0.01). **Table S4**. High confdence modifcation sites identifed in pHASED (p < 0.01). **Table S5**. Unique and common phosphoproteins identifed in LFQ and pHASED datasets. **Table S6**. Phosphorylated master protein kinases identifed in LFQ dataset (p < 0.01). **Table S7**. Phosphorylated master protein kinases identifed in pHASED dataset (p < 0.01). **Table S8**. FLT3-D835 mutations associated with resistance to tyrosine kinase FLT3 inhibitors. **Table S9**. Kinase-Substrate analysis of LFQ dataset for resistant cells in comparison to FLT3-ITD (log2 fold change±0.5). **Table S10**. Kinase-Substrate analysis of pHASED data?set for resistant cells in comparison to FLT3-ITD (log2 fold change±0.5). **Table S11**. Canonical pathways identifed as signifcantly associated with LFQ dataset for resistant cells in comparison to FLT3-ITD. **Table S12**. Canonical pathways identifed as signifcantly associated with pHASED dataset for resistant cells in comparison to FLT3-ITD. **Table S13**. Kinase activity inferred by KSEA analysis of phosphorylation changes in pHASED dataset (log2±0.5, p≤0.05) for resistant cells in comparison to FLT3-ITD. **Table S14**. Mutation-specifc response to sorafenib. IC50 compared to FLT3-ITD. **Table S15**. Bliss Synergy scores for sorafenib in combination with KU-60019 at diferent doses. **Table S16**. Unique ATM substrates identifed with increased phosphorylation (log2≥0.5) in pHASED dataset for resistant cells in comparison to FLT3-ITD. **Table S17**. Vector mutations in FLT3 gene.

## References

[1] Staudt, D.E., Murray, H.C., Skerrett-Byrne, D.A. et al. Phospho-heavy-labeled-spiketide FAIMS stepped-CV DDA (pHASED) provides real-time phosphoproteomics data to aid in cancer drug selection. Clin Proteom 19, 48 (2022). 10.1186/s12014-022-09385-7 10.1186/s12014-022-09385-7 PMC9762002 36536316

