# Supplementary material for: Phospho-heavy-labeled-spiketide FAIMS stepped-CV DDA (pHASED) provides real-time phosphoproteomics data to aid in cancer drug selection
Source: Clin Proteomics. 2022 Dec 19;19:48. doi: 10.1186/s12014-022-09385-7 (PMC9762002; doi:10.1186/s12014-022-09385-7)
Supplement: Supplementary file 1 — Additional file 1. Additional materials and methods. Figure S1. Standard spike-in control abundances (n = 3 biological replicates). Abundances for heavy-labeled spike-in peptides phosphorylated at tyrosine (Y), threonine (T), and serine (S) residues for FLT3-mutant cell lines. A Raw and B normalized peptide abundances of spike-in controls according to different FLT3 cell lines. Total SBDS C raw and D normalized protein abundances for FLT3-ITD, FLT3-ITD/D835V, and FLT3-ITD/D835Y cell lines. [file 12014_2022_9385_MOESM1_ESM.docx]

**ADDITIONAL MATERIALS AND METHODS**

*Plasmid and Vector Construction*

FLT3 mutant constructs were cloned into murine stem cell virus-green fluorescent protein (pMSCV-GFP) vectors, of which templates were a kind gift from Leonie K. Ashman (University of Newcastle, Australia). Internal tandem duplication mutation was generated through the insertion of a 69 bp fragment in FLT3 (FLT3-ITD). Mutagenesis was performed using QuikChange II Site-Directed Mutagenesis Kit (Agilent) as per manufacturer’s instructions, to generate FLT3-ITD/D835V and FLT3-ITD/D835Y double mutant receptors with both -ITD and -D835 mutations (substitution of 3 bp in codon D835 (GAT)). -D835V mutant constructs were generated using “GGATTGGCTCGAGTTATCATGAGTGATTCC” and “GGAATCACTCATGATAACTCGAGCCAATCC” primers, whereas “GGATTGGCTCGATATATCATGAGTGATTCC” and “GGAATCACTCATGATATATCGAGCCAATCC” primers were used to generate -D835Y mutants (Sigma). Vector mutations are listed in Table S17.

*Retroviral transduction*

Viral vectors were transformed using XL-Blue Supercompetent cells (Agilent) and extracted DNA (8 µg) transfected into Phoenix Eco cells (Leonie K. Ashman,University of Newcastle). FLT3 mutant myeloblasts were generated through retroviral transduction on FDC-P1 cells using pMSCV-GFP vectors containing different FLT3 mutant cDNAs or empty vector (EV). Cells were selected using puromycin and transformation efficiency analyzed by Sanger DNA sequencing. Successfully transduced FDC-P1 cells were sorted to generate homogeneous populations of each cell type using GFP protein expression (>80%).

*Sanger Sequencing*

DNA was extracted from FDC-P1 transduced cell lines using the Wizard SV Genomic DNA Purification System (Promega), as per manufacturer’s instructions and quantified using NanoDrop 2000 (Thermo Scientific). 50 ng of DNA were separated for Polymerase chain reaction (PCR) amplification and ran in 1% agarose gel at 100V. DNA was extracted from gel using the Wizard SV Gel and PCR Clean-up System (Promega), as per manufacturer’s instructions, and sent for Sanger sequencing by the Australian Genome Research Facility (AGRF, Australia).

**FIGURE S1**


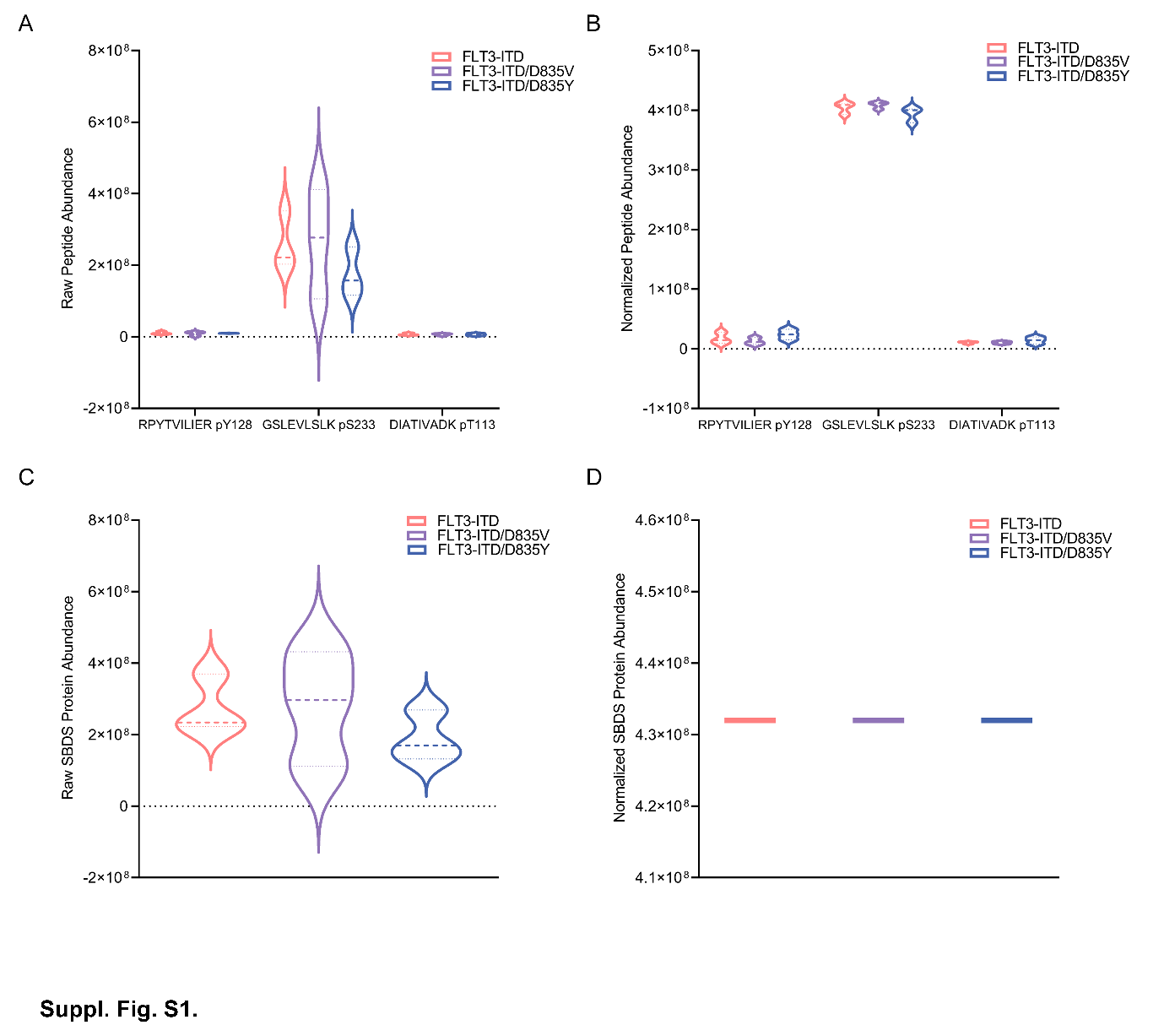


**Figure S1**. **Standard spike-in control abundances (n=3 biological replicates).** Abundances for heavy-labeled spike-in peptides phosphorylated at tyrosine (Y), threonine (T), and serine (S) residues for FLT3-mutant cell lines. *A*) Raw and *B*) normalized peptide abundances of spike-in controls according to different FLT3 cell lines. Total SBDS *C*) raw and *D*) normalized protein abundances for FLT3-ITD, FLT3-ITD/D835V, and FLT3-ITD/D835Y cell lines.
